# Monte Carlo-energy minimization of correolide in the Kv1.3 channel: possible role of potassium ion in ligand-receptor interactions

**DOI:** 10.1186/1472-6807-7-5

**Published:** 2007-01-29

**Authors:** Iva Bruhova, Boris S Zhorov

**Affiliations:** 1 Department of Biochemistry and Biomedical Sciences, McMaster University, 1200 Main Street West, Hamilton, Ontario, L8N 3Z5, Canada

## Abstract

**Background:**

Correolide, a nortriterpene isolated from the Costa Rican tree *Spachea correa*, is a novel immunosuppressant, which blocks Kv1.3 channels in human T lymphocytes. Earlier mutational studies suggest that correolide binds in the channel pore. Correolide has several nucleophilic groups, but the pore-lining helices in Kv1.3 are predominantly hydrophobic raising questions about the nature of correolide-channel interactions.

**Results:**

We employed the method of Monte Carlo (MC) with energy minimization to search for optimal complexes of correolide in Kv1.2-based models of the open Kv1.3 with potassium binding sites 2/4 or 1/3/5 loaded with K+ ions. The energy was MC-minimized from many randomly generated starting positions and orientations of the ligand. In all the predicted low-energy complexes, oxygen atoms of correolide chelate a K+ ion. Correolide-sensing residues known from mutational analysis along with the ligand-bound K+ ion provide major contributions to the ligand-binding energy. Deficiency of K+ ions in the selectivity filter of C-type inactivated Kv1.3 would stabilize K+-bound correolide in the inner pore.

**Conclusion:**

Our study explains the paradox that cationic and nucleophilic ligands bind to the same region in the inner pore of K+ channels and suggests that a K+ ion is an important determinant of the correolide receptor and possibly receptors of other nucleophilic blockers of the inner pore of K+ channels.

## Background

Potassium channels play fundamental roles in physiology by controlling the electrical activity of excitable cells [1]. The pore-forming subunit of K+ channels is formed by four identical or homologous domains symmetrically arranged around the pore axis. Each domain contains a transmembrane outer helix, a membrane-diving P-loop, and a transmembrane inner helix. The P-loop comprises a pore helix, a selectivity-filter region with the potassium channel signature sequence TVGYG, and an extracellular linker to the inner helix. Voltage-gated K+ channels (Kv) also contain large voltage-sensing domains linked to the N-termini of the outer helices. In the X-ray structures of bacterial K+ channels, KcsA [2] and KirBac [3], the cytoplasmic ends of the pore-lining inner helices converge to form a closed activation gate. KcsA co-crystallized with tetrabutylammonium (TBA) trapped in the closed pore shows the ligand's ammonium group near Thr residues of the selectivity filter [4,5]. In the open channels, MthK [6], KvAP [7], and Kv1.2 [8], the inner helices are kinked at a conserved Gly residue and the diverging C-termini form a wide entrance to the inner vestibule. The wide-open pore region of P-loop channels is a target for various open-channel blockers [9].

Numerous naturally occurring and synthetic compounds block Kv channels [10]. Classical low molecular weight blockers such as hydrophobic cations tetraethylammonium and TBA are non-selective drugs, which bind to various subtypes of K+ channels. Low molecular weight blockers that selectively target Kv channels have great potential as pharmaceuticals. One of such drugs is correolide, a nortriterpene alkaloid isolated from the Costa Rican tree *Spachea correa*. Correolide blocks channels of the Kv1 family with higher affinity than other Kv channels [11,12]. Within the Kv1 family, the fastest kinetics of correolide binding is observed for Kv1.3 and Kv1.4 channels [12]. Correolide prevents the activation of T-cells by selectively blocking the open or C-type inactivated Kv1.3 channels [13]. Correolide and its derivatives are candidates for the development of novel immunosuppressant drugs for the treatment of graft rejection and autoimmune diseases [14]. Mapping of correolide receptor in Kv1.3 channel may help design these drugs.

Mutational and ligand-binding studies predicted that dihydrocorreolide (henceforth referred to as correolide) binds in the central pore of Kv1.3 [15]. Earlier we have built the KvAP-based model of the *Shaker *channel, which explained Cd2+-binding experiments [16-18] and seemingly paradoxical observations that large correolide and small Cd2+ ions block the open channel at the same level of the pore [19]. Structure of Kv1.2 [8] confirmed major predictions of the model [19], but demonstrated that the open pore of Kv1.2 is ~1 Å narrower than that in KvAP. The 9 Å-wide pore of Kv1.2 is consistent with the correolide dimensions predicted to be 9 – 10 Å [19]. A recent study shows that another semirigid bulky ligand, d-tubocurarine binds in the open pore of Kv1.3 [20]. Mapping of the correolide receptor in the Kv1.2-based model of Kv1.3 is now warranted to rationalize mutational studies [15] and provide information for possible design of simpler drugs targeting Kv1.3 channels.

**Figure 1:**
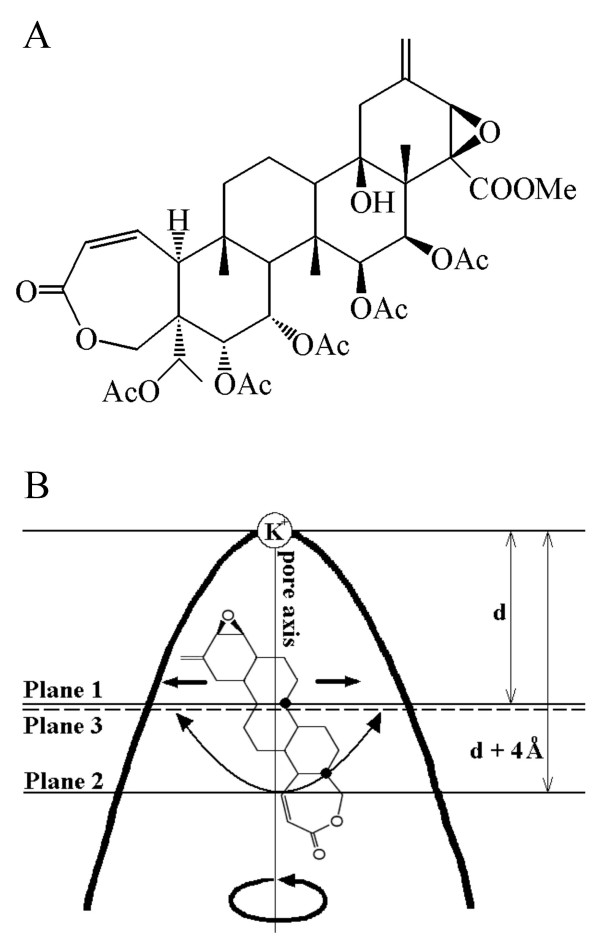
**Structure of correolide**. ***A***, Chemical formula. ***B***, Constraints used to pull correolide along the pore axis in the orientation with the epoxy group towards the selectivity filter. The inner pore is shown schematically by the thick line. K+ ion in position 4 of the selectivity filter is shown as a sphere. A dot-designated driven atom of correolide shared by two 6-membered rings was constrained to plane 1, which is normal to the pore axis. The co-driven dot-designated atom shared by 6- and 7-membered rings was constrained between planes 2 and 3, which are farther from the selectivity filter than plane 1. All three planes were concertedly moved with the step of 0.5 Å normally to the pore axis, and at each step the energy of the complex was MC-minimized. The driven atom retains two of the three degrees of freedom, while co-driven atom retains all three degrees of freedom, but cannot occur ahead of the driven atom. Overall, the ligand retains all internal degrees of freedom and five out of six rigid-body degrees of freedom. The curved arrows show that the correolide can turn around the pore axis and its long axis can decline significantly from the pore axis, but the ligand cannot flip-flop. A similar system of constraints was used to move correolide with its 7-membered ring towards the selectivity filter.

Several theoretical and experimental studies predicted the involvement of metal ions in ligand-receptor interactions in ion channels [21-24]. However, no direct experimental data on the ternary complex formation is yet available. In this regard, the complex of Kv1.3 with correolide seems to be a promising object to further address this problem computationally. Indeed, the large semirigid and nucleophilic ligand (Figure 1A) should adopt a limited number of binding modes in the pore, while the X-ray structures of K+ channels show that the K+ ion bound to Thr residues in the four TVGYG motifs (position 4 according to [25]) may be accessible from the cytoplasmic side by ligands. A metal ion in the focus of macrodipoles of the pore helices (position 5) also may interact with nucleophilic ligands [26].

In this work, we have built Kv1.2-based models of the open Kv1.3 with potassium binding sites 2/4 or 1/3/5 loaded by K+ ions. The respective models are named 2/4 and 1/3/5. We further searched for the energetically optimal positions and orientations of correolide in models 2/4 and 1/3/5 by launching Monte Carlo-energy minimization (MCM) trajectories from a large number of random starting points. To explore whether the bulky correolide can reach the selectivity filter from the cytoplasm, we also computed profiles of MC-minimized energy of the drug pulled through the inner pore of model 2/4. Calculations predict that correolide can bind inside the pore in both models 2/4 and 1/3/5 and chelate a K+ ion in position 4 or 5, respectively. In both 2/4 and 1/3/5 models, most of the experimentally detected correolide-sensing residues directly interact with the drug. A large contribution to the ligand binding energy provided by a potassium ion suggests that it is an indispensable part of the correolide receptor.

## Results

### Correolide structure

The semirigid molecule of correolide seen in the X-ray structure [27] has the shape of a flattened ellipsoid with epoxide oxygen at one pole and carbonyl oxygen in the seven-membered ring at another. Let us define the long axis of correolide as a line drawn between the poles, which are ~12 Å apart. The length of correolide is ~16 Å, which is defined as the distance between the most remote points at the van der Waals surfaces of the opposite poles. The length significantly exceeds the width of the open pore, which is ~9 Å in Kv1.2. This rules out the orientation of correolide with its long axis normal to the pore axis. The molecule contains an epoxide, ester, hydroxyl, acetyl, and five acetoxy groups with a total of 16 oxygen atoms. These groups can accept up to 32 H-bonds and donate only one H-bond. This makes correolide a nucleophilic molecule. However, the nucleophilic potential of the ligand is not matched by the inner vestibule of the channel, which is predominantly lined with hydrophobic residues in the inner helices. Thr^391 ^and Thr^392 ^in the pore helices could provide H-bond donors to few oxygen atoms at the poles of correolide but not to other oxygens. The lack of chemical complementarity between correolide and the inner vestibule rules out the application of ligand-receptor constraints to bias specific orientations of the drug. Therefore, no constraints were used during the random search for the optimal binding modes of the ligand.

### Correolide in the Kv1.2-based model of the open Kv1.3

**Figure 2:**
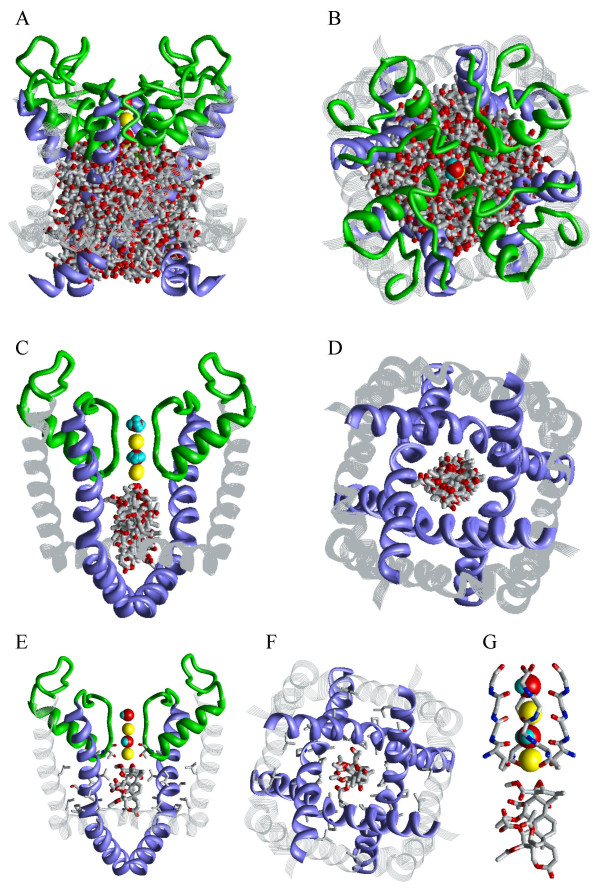
**Random search for the energetically optimal binding site of correolide in model 2/4**. The following coloring scheme is used: inner helices – violet ribbons; pore helices – green ribbons; outer helices – gray strands; the selectivity-filter region and extracellular segments – green rods; K+ ions – yellow spheres; water molecules – space filled; correolide – sticks with gray carbons and red oxygens. ***A ***and ***B***, The side and extracellular views of 200 out of 20,000 randomly generated starting positions of correolide, in which its mass center occurred within a cylinder of 16 Å in diameter and 16 Å in length. ***C ***and ***D***, The side and cytoplasmic views of the superposition of six lowest-energy structures found after energy minimizations from the 20,000 starting points. In the side view, only two domains are shown for clarity. In the cytoplasmic view, the P-loop domain is not shown for clarity. ***E ***and ***F***, The structure with the most favorable ligand-receptor energy, whose characteristics are given in Table 2. Side chains of correolide-sensing residues found by Hanner et al. [15] are shown as sticks. ***G***, Close-up view of the complex shown at ***E***. Note that two oxygen atoms of correolide, one of which from the epoxide group, bind to the K+ ion in position 4, which is also coordinated by eight oxygen atoms from residues Thr^392^.

The Kv1.2-based homology model of the pore domain of Kv1.3 contains outer helices, P-loops, and inner helices (Table 1). The voltage-sensing domains were not modeled. To predict the energetically optimal binding modes of correolide inside the 2/4 model of Kv1.3, 20,000 positions and orientations of the ligand were randomly generated within a cylinder of 16 × 16 Å (Figure 2A, 2B). From each starting point, the energy was minimized. A thousand of the lowest-energy conformations found at this stage were further MC-minimized. Six structures within 5 kcal/mol from the apparent global minimum show that correolide can adopt various positions and orientations inside the pore (Figures 2C, 2D). The random search did not predict any low-energy complexes with the ligand in the interface between domains. In the lowest-energy complexes found, correolide interacts with the K+ ion in position 4, which is coordinated between the side chains and backbone oxygens of Thr^392 ^(Figures 2E, 2G). Energy characteristics of the representative complexes in which correolide chelates the K+ ion by either the ether group in the seven-membered ring or the epoxy group are given in Table 2. The complexes are stabilized by van der Waals and electrostatic interactions, but do not contain intermolecular H-bonds. Table 1 highlights nine inner-helix residues, whose mutations affect correolide binding [15]. Seven of these residues provide contributions to the ligand-receptor energy ≥ |0.4| kcal/mol (Table 2). In addition, Thr^391 ^and Thr^392 ^at the selectivity filter also interact with the drug, but the threonines from different domains provide either favorable or unfavorable contributions, which counterbalance each other. In the optimal complexes, oxygen atoms at the poles of the ellipsoid-shaped correolide form direct contacts with the K+ ion in position 4, which contributes up to -4.6 kcal/mol to the ligand-receptor energy. Several other oxygen atoms of correolide also contribute stabilizing electrostatic energy by interacting with K+ ions.

**Table 1:** Sequence of Kv1.3*

	Residue #	
Outer Helix	331	GLQILGQTLK ASMRE**L**GLLI FF**L**FIGVILF SSAVYFAE
P-loop	376	FSSIPDAFWW AVVTMTTVGY GDMHPVT
Inner Helix	403	IGGKIVGSLC **A**IAG**VLT**I**AL PVP**VIVSNFN YFYH

* Bold-typed are residues whose mutations change correolide binding energy by more than 1 kcal/mol [15].

**Table 2:** Correolide-sensing residues in the inner helices^a ^and their energy contributions ^b ^(kcal/mol) to correolide binding

	K+-chelating groups of correolide				
	
	Model 2/4		Model 1/3/5		
	
	Epoxy	Ether^c^	Epoxy	Ether^c^	Acetoxy^d^
Inner-helix mutations affecting correolide binding ^a^					
					
Ala^413^Cys					
Val^417^Ala	-2.6	-3.1	-2.3	-2.6	-2.5
Leu^418^Ala	-1.4	-0.9	-1.8	-1.9	-1.7
Thr^419^Ala			-0.6	-0.4	
Ala^421^Cys	-4.2	-2.5	-1.8	-5.0	-2.0
Leu^422^Ala	-0.9	-0.4	1.2	-2.1	-0.6
Pro^423^Ala		-1.0		-1.2	
Val^424^Ala	-2.2	-4.7	-4.2	-2.8	-4.5
Pro^425^Ala	-1.0	-4.4	-3.7	-1.7	
					
Mutation affecting channel expression ^e^					
Ile^420^Ala	-5.1	-1.3	-0.6	-0.8	-1.8
					
Predicted ligand-receptor energy	-20.6	-20.3	-19.1	-22.4	-21.9

^a ^Mutations that change correolide binding energy by more than 1 kcal/mol [15].

^b ^Energy contribution of a residue was summed over four domains. Contributions with the absolute energy less than 0.4 kcal/mol are not shown.

^c ^Ether group in the seven-membered ring.

^d ^Three acetoxy groups (Fig. 4E).

^e ^Ile^420 ^contributes to correolide binding in our models, but is not categorized as a correolide-sensing residue because its mutation results in poor expression of the channels [15].

**Figure 3:**
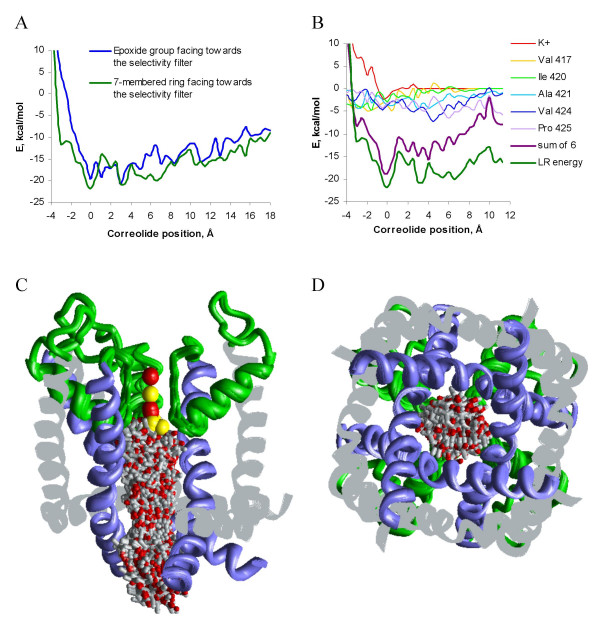
**Systematic search for the energetically optimal binding site of correolide in model 2/4**. ***A***, Ligand-receptor energy of correolide pulled through the pore. The zero translational position is calibrated to show the complex with an oxygen atom of correolide bound to the K+ ion in position 4. Blue and green lines represent trajectories with the epoxy group oriented towards and away from the selectivity filter, respectively. ***B***, Contributions of K+ ions and correolide-sensing residues revealed by Hanner et al. [15] to the interaction energy of correolide oriented with its epoxy group away from the selectivity filter. Also included is the contribution of Ile^420 ^whose mutation to Ala affected the channels expression. The contributions of amino acids are summed over four subunits. The energy values are partitioned from the MC-minimized structures at specific translational positions. In most of the translational positions around the selectivity filter, the sum of six monitored contributions is close to the total ligand-receptor energy indicating that the contribution of other residues is small. At the cytoplasmic entrance to the open pore, residues Val^428 ^and Asn^432 ^contribute energy to correolide binding. ***C ***and ***D***, The side and cytoplasmic views of superposed MC-minimized complexes at different translational positions with the 7-membered ring facing the selectivity filter. Displacement of the K+ ion from the pore axis occurs at the high-energy leftmost points of the profile (***A***, ***B***) where correolide is forced into the selectivity filter.

While docking correolide from many randomly generated starting points predicts energetically preferable binding modes, it does not allow concluding whether the binding site is reachable for correolide from the cytoplasm. Indeed, *a priori*, we could not rule out that a large energy barrier may preclude access of the bulky correolide molecule to the selectivity-filter region of Kv1.3. To address this problem, we pulled the ligand through the pore in two different orientations: with either the epoxy group or the seven-membered ring oriented towards the selectivity filter. The translational trajectories were 25 Å long to ensure a thorough sampling of the space between the cytoplasmic entry to the pore and the selectivity filter. During this search, a flat-bottom atom-plane constraint was imposed on the K+ ion in position 4 to allow its penalty-free displacement up to 2 Å from the level defined in the X-ray structure. Further displacements were restrained by the penalty of 10 kcal mol^-1 ^Å ^-1^. Figure 3A shows the correolide-channel energy, which was partitioned from the MC-minimized structures. As correolide moves inside the channel and makes an increasing number of favorable contacts, the ligand-receptor energy decreases (becomes more favorable). The energy reaches the minimum as the ligand binds to the K+ ion in position 4, which is coordinated by residues Thr^392 ^in the selectivity filter. Further advancement of correolide results in the energy increase due to repulsion from Thr^392^. The plots of ligand-receptor energy against correolide position in the pore do not show large energy barriers with both orientations of the drug (Figure 3A) indicating that the binding site at the selectivity filter is reachable by correolide from the cytoplasm.

As correolide approaches a residue, the stabilizing contribution of the residue to the ligand-receptor energy increases (Figure 3B). No inner-helix residue contributes positive energy to ligand-receptor interactions, indicating that unfavorable contacts, which are unavoidable in the starting conformations, have been relaxed in MC-minimizations. The plot of partitioned ligand-receptor energy (Figure 3B) shows that K+ ion in position 4 contributes ~-2.5 kcal/mol to ligand-receptor energy. This energy is weaker than the contribution of -4.6 kcal/mol found during the random search. This is because the ligand, which is constrained to the plane normal to the pore axis at each point of the profile, cannot establish optimal interactions with the ion. As correolide approaches the selectivity filter, the total contribution of the K+ ion and the pore-facing correolide-sensing residues identified in mutational experiments [15] is close to the entire ligand-receptor energy (Figure 3B). Leu^346 ^and Val^428 ^stabilize correolide at the entry to the inner pore. Interestingly, Leu^346 ^was detected as a correolide-sensing residue in mutational experiments [15].

**Figure 4:**
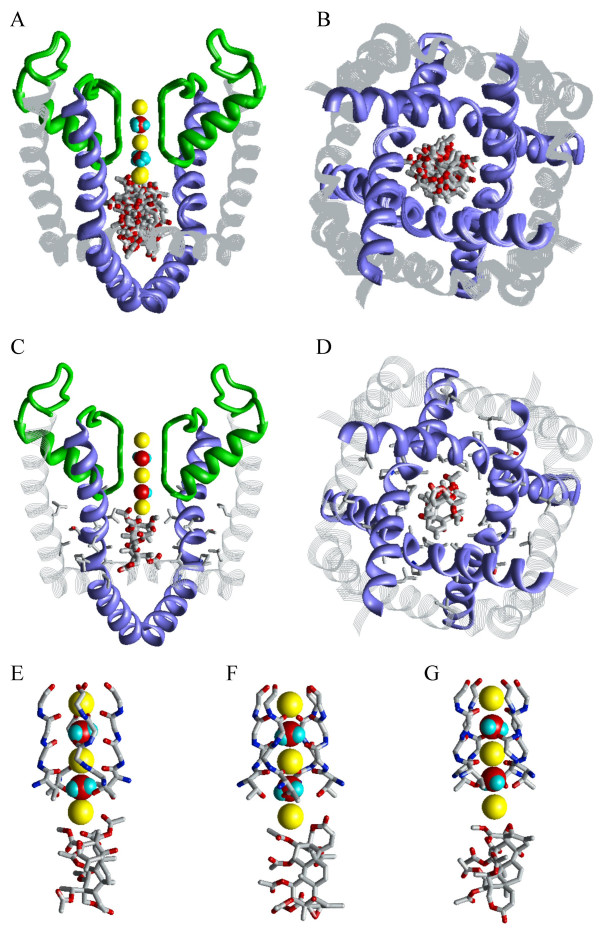
**Correolide in model 1/3/5 of Kv1.3**. ***A ***and ***B***, The side and intracellular views of 12 energetically best structures obtained from 20,000 randomly generated starting positions of correolide, in which its mass center occurred within a cylinder of 16 Å in diameter and 16 Å in length. ***C ***and ***D***, The lowest-energy binding mode of correolide with 3 acetoxy groups chelating K+ ion in position 5. Side chains of correolide-sensing residues found by Hanner et al. [15] are shown as sticks. ***E-G***, The close-up view at structures with the most favorable ligand-receptor energy, whose characteristics are given in Table 2. The K+ ion in position 5 is chelated by three acetoxy groups (***E***), an acetoxy group and the ether group from the seven-membered ring (***F***), and epoxy and acetyl groups (***G***).

A metal ion in the focus of macrodipoles of the pore helices (position 5) was recently proposed to play a crucial role in the binding of benzocaine to sodium channels [26]. To explore a similar possibility in Kv1.3, we performed a random search of correolide binding modes in model 1/3/5. Representative low-energy structures are shown in Figure 4 and their energy characteristics are given in Table 2. Since K+ ion in position 5 does not interact directly with the channel residues, a large part of its surface is available for chelation by the ligand. Indeed, an interesting binding mode was found with three acetoxy groups chelating the K+ ion (Figure 4C–E). In this mode, neither epoxy group nor seven-membered ring interacts with K+. Such a binding mode may explain why elimination of the epoxy group and removal of the carbonyl oxygen from the seven-membered ring does not abolish the channel-blocking activity of correolide [28]. The same correolide-sensing residues that contribute to correolide binding in model 2/4 also contribute to the ligand binding in model 1/3/5 (Table 2). The ligand-receptor energies in model 1/3/5 are only 1 – 2 kcal/mol more preferable than in model 2/4 (Table 2). The small energy difference does not allow us to favor model 1/3/5 over model 2/4. Furthermore, we cannot rule out that both binding modes may coexist.

Thus, our calculations predict several binding modes of correolide in Kv1.3. The population of these modes would depend on the pattern in which K+ binding sites are occupied by K+ ions and water molecules. When position 4 is occupied by K+, both the random and systematic MCM search predict the selectivity-filter region to be an important structural determinant of the correolide receptor (Figures 2 and 3). When position 5 is occupied by the K+ ion, correolide would readily bind it, providing up to three oxygens to the K+ coordination sphere (Figure 4).

### Sensitivity of results to the chosen computational methodology

The goals of this study were to predict the binding site for correolide and to explore whether the K+ ion could contribute to the correolide receptor. To perform the extensive search for the lowest-energy complexes between Kv1.3 and correolide, we used several approximations: rather small cutoff of 8 Å, implicit solvent, simple treatment of the electrostatic interactions, and neutral forms of ionizable residues. Such approximations are hardly acceptable in computational studies aimed to predict the free energy of ligand binding or simulate ion permeation. However, results of the correolide receptor mapping are less critical to the method of energy calculation. Indeed, in the best complexes, correolide appears to fit in the inner pore. The geometry of the tight ligand-channel complex is defined primarily by the van der Waals energy, which is reliably predicted with different force fields. Nevertheless, to assess the sensitivity of our results to variations in methodological setup, we reevaluated the geometry and energy of the correolide complex with model 2/4 by submitting additional MCM trajectories starting from the optimal structure predicted in the random search (Figure 2). The additional MCM trajectories were run with a larger cutoff, ionized titrable residues, weaker electrostatics, and K+ ion removed from position 2.

**Figure 5:**
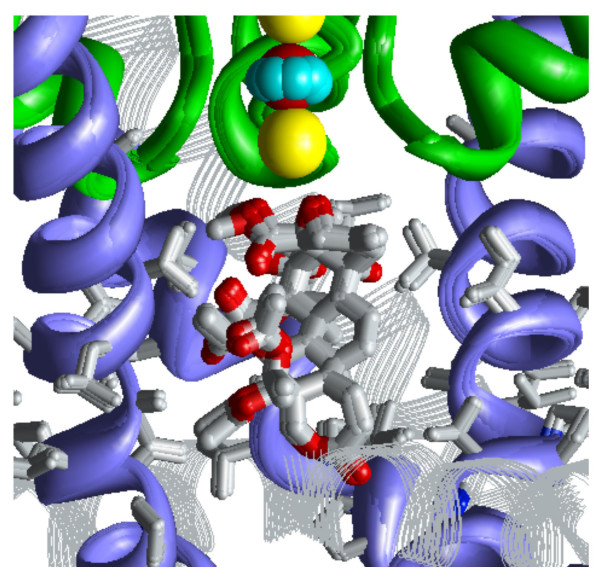
**Kv1.3:correolide models calculated with different settings**. Superimposed models 2/4 calculated with different methodological settings (Table 3). Correolide faces the selectivity filter with its epoxy group. Correolide-sensing residues are shown as sticks. One domain is removed for clarity.

Results show that the geometry of the MC-minimized structures remains practically unchanged under different methodological setups (Figure 5). Among the different methodological settings that could potentially affect the ligand-receptor energy, only reduced electrostatic interactions weakened the ligand-receptor energy, which nevertheless remained preferable (Table 3). The involvement of a K+ ion in correolide binding is the most important prediction, which is insensitive to the variations of methodology. Indeed, correolide does not bring charged or ionizable groups to the selectivity filter, but just replaces one to three water molecules from the cytoplasmic face of a K+ ion at the pore axis.

**Table 3:** Ligand-receptor energy predicted with various methodological setups ^a^

Variations in the methodological setup ^b^	Ligand-receptor energy (kcal/mol)
Standard protocol (see methods)	-20.6
Cutoff, 12 Å	-22.0
Electrostatics, ε = 2 d	-13.6
1,4 occupancy of selectivity-filter by K+ ions	-22.1
Ionized titrable residues	-23.4

^a ^Calculations were performed in the Kv1.2-based model 2/4 with correolide bound with the epoxy group towards the selectivity filter. Each value of the ligand-receptor energy was obtained in an MCM trajectory that started from the best structure found by the random search (Figure 2E) and terminated when the last 1000 energy minimizations did not decrease the energy of the apparent global minimum found.

^b ^Only deviations from the standard protocol are indicated.

## Discussion

Various naturally occurring and synthetic compounds targeting Kv channels have been characterized [10]. Classical blockers of K+ channels such as tetraethylammonium and peptidyl toxins lack selectivity to different subtypes of K+ channels. Small-molecule blockers selectively targeting specific Kv channels are valuable tools for basic studies and have large potential as pharmaceuticals. *Shaker*-type Kv1.3 channels that control membrane potential and calcium influx are important targets for drug discovery. Correolide is the first small-molecule ligand isolated from a natural product, which blocks Kv1.3 channels in T cells. Understanding the mechanism of correolide block could help develop other immunosuppressants as well as selective blockers of various Kv channels.

Little structural information is available on the complexes of small-molecule ligands with P-loop channels. The crystallographic structure of a ligand-bound KcsA [4,5] shows TBA trapped in the water-lake cavity with the center of the ammonium group being near to the focus of four macrodipoles of the pore helices. Unlike TBA, correolide is an electrically neutral ligand with numerous nucleophilic groups. Mutational studies [15] revealed correolide-sensing residues in the inner and outer helices of Kv1.3, but did not explain the causes of high-affinity binding of the drug. Furthermore, all correolide-sensing residues revealed in study [15] cannot bind simultaneously to the drug in any reasonable model of the ion channel. Therefore, the three-dimensional mapping of the correolide receptor was one of the aims of our study.

### Correolide-sensing residues

**Figure 6:**
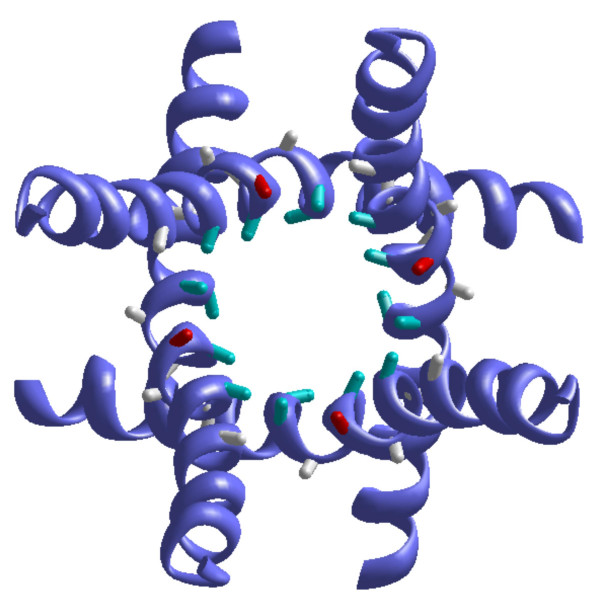
**The extracellular view at the inner-helices bundle in the open Kv1.3**. Bonds C^α^-C^β ^in correolide-sensing residues [15] are shown as either cyan sticks that face the pore or gray sticks directed away from the pore. Red sticks show bonds C^α^-C^β ^in Ile^420^, the residue whose mutation to Ala results in low expression of Kv1.3 [15]. Four correolide sensing residues face the pore, while Ile^420 ^could stabilize the channel conformation by interacting with the outer helices, which are not shown.

Hanner et al. [15] revealed nine residues in the inner helices, whose mutation changes correolide binding energy by more than 1 kcal/mol (Table 2). Five of these residues face the inner pore (Figure 6) and provide noticeable energy to correolide binding in both 2/4 and 1/3/5 models of Kv1.3 (Table 2). Why the remaining four residues in the inner helices affect correolide binding in experiments but not in the model? One of these residues is Ala^413 ^whose substitution with Cys affects correolide binding in mutational experiments [15]. However, Ala^413 ^does not contribute to correolide-binding energy (Table 2). The cause may be that Ala^413 ^approaches Val^393 ^in the selectivity filter. The substitution of Ala^413 ^with a larger Cys may affect the selectivity filter structure and interaction of correolide with a K+ ion at the selectivity filter. Ogielska and Aldrich [29] found that the Ala^413^Cys mutation in Kv1.3 decreases the affinity for K+ ions, possibly due to conformational changes at the selectivity filter. In view of our model, these data support the notion that a K+ ion at the selectivity filter can stabilize correolide binding.

The mutation of Pro^423 ^in the PVP motif of the inner helix affects correolide binding, however Pro^423 ^does not provide noticeable contribution to the ligand-receptor energy in three ligand-binding modes characterized in Table 2. The substitution of Pro^423 ^could decrease the flexibility of the inner helix by enabling the backbone NH group in this position to form an H-bond with carbonyl oxygens in positions 419–420. This may change the orientation of Val^424 ^and Pro^425 ^residues that affect correolide binding in both experiments and computational models (Table 2).

According to our model, Ile^420 ^contributes to correolide binding (Table 2). However, experimental data on the involvement of Ile^420 ^in correolide binding are not available. The mutation Ile^420^Ala results in the low expression of Kv1.3 [15] indicating that large hydrophobic Ile^420 ^is involved in the stabilization of the channel structure. Such stabilization is more likely if the side chains of Ile^420 ^interact with other transmembrane helices rather than face the pore. Indeed, in the Kv1.2-based model, Ile^420 ^is exposed to the inter-segment interface (Figure 6).

### Possible involvement of K+ in correolide binding

Several models of Ca2+ and Na+ channels with the pore-bound nucleophilic ligands suggest that the permeable metal ions may contribute to ligand-receptor complexes [22-24,26]. Recent experiments addressing the mechanism of action of batrachotoxin in the Na_v_1.4 channel [30] confirmed important predictions of the ternary-complex model. However, the direct experimental validation of the ternary-complex concept is still difficult. The major problems are the uncertain location of the metal ions, relatively low stability of their complexes with the channels, conformational flexibility of drugs, and unknown location of their binding sites. Correolide seems to be an appropriate ligand to investigate the possibility of its ternary association with the receptor and K+ ion because of four reasons. First, the drug has a semirigid conformation that would not change significantly upon the binding to the channel and/or to the ion. Second, the size of correolide is compatible with the size of the open pore [9,19], thus decreasing the uncertainty of the binding-site location. Third, correolide has an ellipsoidal shape with an epoxy group at one pole and ester group at another pole (Figure 1A). These groups can accept but not donate H-bonds and they can interact with metal ions. The nucleophilic character of correolide and the predominantly hydrophobic character of the inner helices in Kv1.3 suggest that a K+ ion in position 4 or 5 may provide an electrostatic component for the drug-receptor energy. Fourth, approximate locations of K+ ions in potassium channels are known from experiments. Importantly, in this study multiple positions and orientations of correolide in the channel were intensively sampled to avoid any bias on the ternary association of the drug with the channel and K+. The results suggest that the ternary complexes can explain peculiarities of correolide structure, results of mutational analysis of correolide binding, as well as coupling of correolide- and K+ binding sites [11].

### Possible role of C-type inactivation in correolide binding

Correolide binds to Kv1.3 and Kv1.4 channels with a higher affinity than to other channels of the Kv1 family [12]. Since the inner and outer helices are conserved in Kv1 channels, correolide-sensing residues in these helices are unlikely to determine correolide selectivity to Kv1.3 and Kv1.4. What distinguishes the latter channels is C-type inactivation, which is less pronounced in other members of the Kv1 family. How could C-type inactivation enhance correolide binding? A recent study suggested that C-type inactivation might be caused by the rearrangement of the selectivity filter in a way that the K+ in position 4 remains the only cation in the selectivity-filter region [5]. Our models 2/4 and 1/3/5 predict the strong involvement of a K+ ion in correolide binding. In C-type inactivated channels, a deficiency of K+ ions in positions 1 – 3 would stabilize the K+-bound correolide. Some analogy may be found in a ligand containing an ionizable amino group. When a proton binds to the group, the proton-ligand complex is considered as a protonated ligand, even when the proton is shared with a nucleophilic group of the receptor. Similarly, when a ligand binds K+, the complex may be considered as a K+-containing ligand that would bind stronger to the channels, in which potassium-binding sites 1 – 3 are not occupied by K+ ions. This can explain the intriguing observations that the C-type inactivation enhances binding of both cationic and nucleophilic ligands in the inner pore of K+ channels.

## Conclusion

In this study we predicted the ternary complex between K+, correolide, and K+ channel and suggested a mechanism by which C-type inactivation could enhance correolide binding. The analysis of structure-activity relationships of open-channel blockers of K+ channels shows that many blockers have nucleophilic groups whose role seems unclear given the rather hydrophobic structure of the open pore. Our study suggests that such groups can bind to the channel-bound K+ ions, which may be important determinants of corresponding receptors.

## Methods

The sequence of the α subunit of the human Kv1.3 channel was taken from the SwissProt database (code CIK3_HUMAN). Homology model of the pore domain of Kv1.3 that incorporate the outer helices, P-loops, and the inner helices (Table 1) were built using methodology described elsewhere [20]. The X-ray structure of Kv1.2 (Protein Data Bank code 2A79) was used as the template. All-*trans *starting conformations were assigned for those side chains that were not resolved in the crystal structures. The X-ray structure of correolide [27] was used a starting approximation.

Energy calculations were performed with the ZMM program [31]. Atom-atom interactions were calculated using the AMBER force field [32] with an 8 Å cutoff. The optimal conformations were searched by the MCM method [33]. Hydration energy was calculated using the implicit-solvent method [34]. Hydration of the membrane-exposed residues of the outer helices is a methodological inadequacy. However, this does not affect results of correolide docking in the inner pore because the lipid-facing residues are rather far from the ligand, while the channel folding remained unchanged in this work. Parameters for K+ hydration were chosen to be the same as those for a NH_3_+ group. Electrostatic energy was calculated using the distance-dependent dielectric ε = d [32]. All ionizable residues in the pore domain of Kv1.3 are located at the water-accessible intracellular and extracellular faces, far from correolide-sensing residues identified experimentally [15]. Since these residues may be counterbalanced by counterions, they were considered in their neutral (non-ionized) forms, the approach used in other studies with the implicit solvent [34,35]. Correolide atomic charges were calculated by the AM1 method [36] using MOPAC. Both torsional and bond angles of correolide were allowed to vary during energy minimizations. The Kv1.2-based model was initially MC-minimized starting from the X-ray structure. Following Zhou and MacKinnon [25], the binding sites for K+ in the selectivity-filter region are numbered from 1 to 5 starting from the most extracellular site. In model 2/4, potassium binding sites 2 and 4 were loaded by K+ ions and sites 1 and 3 by water molecules. In model 1/3/5, potassium binding sites 1, 3, and 5 were loaded by K+ ions and sites 2 and 4 by water molecules. The above waters in potassium binding sites were the only explicit water molecules in our calculations.

The optimal positions and orientations of correolide were searched by random and systematic approaches. In the first approach, many MCM trajectories were launched starting from randomly generated positions and orientations of the drug. The area of the random search covered the entire pore region, including interfaces between domains. The systematic search was performed by computing profiles of MC-minimized energy for the drug pulled along the pore axis [37]. Two atom-plane constraints were imposed to allow the ligand's long axis to decline up to 90° to the pore axis, but retain the orientation of the given pole towards the selectivity filter, while the opposite pole faced the cytoplasm. For a given translational position, the driven atom was constrained to a plane normal to the pore axis and the co-driven atom between two planes normal to the pore axis (Figure 1B). The three planes were translated simultaneously along the pore axis with a step of 0.5 Å, and at each step the energy was MC-minimized.

Each MCM trajectory of the correolide-channel complex was computed in two stages. In the first stage, the protein backbone and K+ ions were fixed and energy was MC-minimized with varying protein side chains and all degrees of freedom in the ligand until the last 1000 energy minimizations did not improve the best minimum found. In the second stage, all degrees of freedom were allowed to vary, while the protein alpha carbons were constrained to the template positions using pins. A pin is a flat-bottom parabolic penalty function that allows penalty-free deviation of an atom up to 1 Å from the corresponding position in the X-ray structure of the template and applies the force of 10 kcal mol^-1 ^Å ^-1 ^for further deviations. The second MCM trajectories were terminated when the last 1000 consecutive energy minimizations did not decrease the lowest energy found.

## Abbreviations

Kv, voltage gated potassium channels; Kv1.2 and Kv1.3, subtypes of the *Shaker *potassium channels; 2/4, Kv1.3 model in which potassium binding sites 2 and 4 are loaded with K+ ions; 1/3/5, Kv1.3 model in which potassium binding sites 1, 3, and 5 are loaded with K+ ions; KcsA, MthK, KvAP, and KirBac, bacterial potassium channels; Na_v_1.4, a voltage-gated sodium channel; MC, Monte Carlo; MCM, Monte Carlo with energy minimization; TBA, tetrabutylammonium.

## Authors' contributions

IB performed the research, in particular, built and optimized molecular models, docked correolide, analyzed results, created figures, and participated in the manuscript preparation. BSZ planned and supervised the research, analyzed results, and wrote the manuscript. Both authors read and approved the final manuscript.
